# Synergistic Effects of Erythromycin and Budesonide on the Mitigation of Tracheal Stenosis Associated With Macrophage Phenotype Alteration and HDAC2 Upregulation

**DOI:** 10.1155/carj/8760537

**Published:** 2026-06-23

**Authors:** Jinghua Gan, Guangnan Liu

**Affiliations:** ^1^ Department of Respiratory and Critical Care Medicine, The Second Affiliated Hospital of Guangxi Medical University, Nanning, China, gxmu.edu.cn

**Keywords:** budesonide, erythromycin, histone deacetylase 2 (HDAC2), macrophage polarization, tracheal stenosis (TS)

## Abstract

**Objective:**

We investigated the therapeutic effects of combined erythromycin and budesonide treatment on tracheal stenosis (TS) and the associated changes in macrophage‐related markers and HDAC2 expression.

**Methods:**

A rabbit model of TS was established by mechanical scraping of the tracheal inner wall, and the animals were then treated with erythromycin, budesonide, or their combination. Treatment effects were evaluated through histological analysis. We quantified the mRNA expression of M1 and M2 macrophage markers using quantitative PCR and assessed the protein expression of histone deacetylase 2 (HDAC2) by western blotting. RAW264.7 cells were stimulated with lipopolysaccharide or interleukin‐4, and macrophage phenotypic changes after erythromycin and budesonide treatment were assessed by flow cytometry. M1/M2 macrophages were co‐cultured with fibroblasts, and alpha smooth muscle actin (α‐SMA) expression was detected by immunofluorescence.

**Results:**

The rabbit TS model exhibited TS characterized by significant thickening of the tracheal mucosa and submucosa. These histological changes were ameliorated in the treatment groups, with the greatest improvement observed in the combination therapy group. The mRNA expression levels of iNOS, CD206, CD163, and Arg1, as well as the protein expression of HDAC2, were elevated in treated groups. In vitro, erythromycin combined with budesonide altered macrophage phenotypic distribution, with the M2/M1 ratio shifting toward 1.0. Additionally, fibroblast *α*‐SMA expression varied according to macrophage phenotype in the co‐culture system.

**Conclusion:**

Erythromycin combined with budesonide effectively ameliorated injury‐induced TS and was associated with changes in macrophage‐related markers and increased HDAC2 expression.

## 1. Introduction

Tracheal stenosis (TS), a common complication following tracheotomy or prolonged endotracheal intubation, is frequently encountered in respiratory departments and intensive care units [1]. Although interventional airway management provides immediate clinical benefits for patients with pulmonary, airway, or thoracic diseases, procedure‐related injuries such as post‐intubation TS remain a significant concern. The reported incidence of TS varies, ranging from 0.6% to 21% post‐tracheotomy and from 6% to 21% post‐intubation [2].

The molecular basis of TS has not been fully clarified. Histopathological examination of affected tissues, such as tracheal granulomas, has revealed fibroblast activation, excessive collagen deposition, extracellular matrix accumulation, and inflammatory cell infiltration, all of which contribute to local tissue remodeling [3]. However, the precise pathogenic role of inflammation in TS remains unclear. Studies have identified a significant infiltration of macrophages within stenotic airway tissues, accompanied by an imbalance in M1/M2 macrophage polarization [4]. Macrophages play important roles in immune defense, tissue repair, fibrosis, and regeneration. Therefore, a better understanding of macrophage phenotypic regulation during TS may provide useful insight into its pathogenesis and potential therapeutic intervention.

Erythromycin has been reported to inhibit inflammation and tracheal granuloma formation following tracheal injury, thereby contributing to the prevention and treatment of TS [3]. However, the underlying mechanisms remain unclear. Erythromycin is known for its antibacterial properties, as well as its anti‐inflammatory and immunomodulatory effects. The aim of this study was to investigate macrophage‐related changes in TS using animal and cellular models. We further evaluated the effects of erythromycin, alone or in combination with budesonide, on macrophage‐associated markers and tracheal injury‐related changes. These findings may provide additional evidence to support combination therapy for TS.

## 2. Materials and Methods

### 2.1. Experimental Materials

Fifty adult New Zealand white rabbits (25 males and 25 females), weighing 2.0–2.5 kg, were obtained from the Medical Animal Experimental Center of Guangxi Medical University (Nanning, China). All animal procedures were conducted in the university’s accredited animal facility. Erythromycin (#RR820A; Sigma‐Aldrich, St. Louis, MO, USA) and budesonide (#RR820A; AstraZeneca, Cambridge, UK) were prepared according to the manufacturers’ protocols. The following antibodies were used: anti‐histone deacetylase 2 (HDAC2; #RR820A, Cell Signaling Technology, Danvers, MA, USA), anti‐β‐actin (Proteintech, Rosemont, IL, USA), anti‐α‐smooth muscle actin (α‐SMA; Abcam, Cambridge, MA, USA), anti‐F4/80 (#11–4801–82; Invitrogen, Carlsbad, CA, USA), anti‐CD206 (#17–2061–82; Invitrogen), and anti‐inducible nitric oxide synthase (iNOS; #12–5920–82; Invitrogen).

### 2.2. Establishment of the TS Animal Model

The rabbits were randomly assigned to five groups (*n* = 10 per group): control (C), model (M), erythromycin (E), budesonide (B), and erythromycin plus budesonide (EB). TS was induced in groups M, E, B, and EB as previously described [4, 5]. Briefly, under anesthesia, a tracheotomy was performed, and the tracheal mucosa was mechanically abraded using a nylon brush (20 strokes per day) for 10 consecutive days to generate benign stenotic lesions. Group C underwent sham surgery without brushing. Starting on the day of surgery, Group M received daily intragastric saline; Group *E* received erythromycin (15 mg kg^−1^ day^−1^, intragastric); Group B received nebulized budesonide (50 µg kg^−1^ day^−1^); and Group EB received both erythromycin (15 mg kg^−1^ day^−1^, intragastric) and nebulized budesonide (50 µg kg^−1^ day^−1^), following an established protocol [6]. All rabbits were euthanized on day 10 post‐operation, and tracheal segments from the stenotic region were collected for analysis.

### 2.3. Assessment of TS Severity

TS was quantified morphometrically. Transverse sections from the stenotic tracheal segment were harvested. The longest (r1) and shortest (r2) inner diameters of the tracheal lumen, as well as the corresponding longest (R1) and shortest (R2) outer diameters defined by the tracheal cartilage ring, were measured. The percentage of stenosis (S) was calculated using the following formula: *S* = [1 − (r1 × r2)/(R1 × R2)] × 100%, where r1 and r2 represent the longest and shortest inner diameters of the tracheal lumen, respectively, and R1 and R2 represent the corresponding longest and shortest outer diameters defined by the tracheal cartilage ring.

Additionally, the thickness of the combined mucosa and submucosa layer was assessed. With the center of the anterior tracheal wall defined as the 12 o’clock position, measurements were taken at the 12, 3, and 9 o’clock positions using a light microscope (CKX53; Olympus, Tokyo, Japan).

### 2.4. Histological Evaluation Using Hematoxylin and Eosin and Masson’s Trichrome Staining

Tracheal tissue samples were fixed, paraffin‐embedded, and sectioned. Histological assessment was performed using hematoxylin and eosin (H&E) staining to evaluate general morphology and Masson’s trichrome staining to assess collagen deposition, according to the manufacturer’s protocol (G1120; Solarbio Life Science, Beijing, China). To quantify fibrosis, the collagen fiber content within granulation tissue was assessed. For each Masson’s trichrome‐stained slide, 10 nonoverlapping fields of view (200 ×  magnification) were randomly selected. The area positive for collagen (blue staining) within the tracheal epithelial layer and lamina propria was measured using image analysis software.

### 2.5. Real‐Time PCR

Total RNA was isolated from tracheal granulation tissue using the RNA Iso Plus (Takara Bio, Shiga, Japan). RNA concentration and purity were determined spectrophotometrically. First‐strand cDNA was synthesized from 1 µg of total RNA using the PrimeScript RT reagent kit (RR036A; Takara Bio, Shiga, Japan) according to the manufacturer’s protocol. Quantitative PCR was performed in sextuplicate using TB Green Premix Ex Taq II (RR820A; Takara Bio) on an ABI 7500 Fast Real‐Time PCR System (Applied Biosystems, Foster City, CA, USA). The primer sequences used were:

iNOS: Forward 5′‐ACG TGC GTT ACT CCA CCA ACA‐3′, Reverse 5′‐CAT AGC GGA TGA GCT GAG CA‐3′.

CD206: Forward 5′‐CAT ATC GGG TTG AGC CAC TT‐3′, Reverse 5′‐GAG GGA TCT CCT GTG TTC CA‐3′.

CD163: Forward 5′‐TTT GTC AAC TTG AGT CCC TTC AC‐3′, Reverse 5′‐TCC CGC TAC ACT CGT TTT CAC‐3′.

Arg1: Forward 5′‐GCC AAG TCC AGA ACC ATA GG‐3′, Reverse 5′‐CAC AAG CAG ACC AGC CTT TC‐3′.

β‐actin: Forward 5′‐GGG CAC CCA GCA CAA TGA A‐3′, Reverse 5′‐CTA AGT CAT AGT CCG CCT AGA AGC A‐3′.

The thermal cycling conditions comprised an initial denaturation at 95°C for 30 s, followed by 40 cycles of 95°C for 5 s and 60°C for 30 s. Relative mRNA expression levels were calculated using the 2−ΔΔCt method, with *β*‐actin as the endogenous control.

### 2.6. Western Blotting

Total protein was extracted from tracheal granulation tissue using a Nuclear Protein Extraction Kit (R0050; Solarbio, Beijing, China) according to the manufacturer’s instructions. Protein concentrations were determined using the BCA assay. Equal amounts of protein (e.g., 30 µg per lane) were separated by 12% sodium dodecyl sulfate‐polyacrylamide gel electrophoresis (SDS‐PAGE) and subsequently transferred onto polyvinylidene difluoride (PVDF) membranes. After blocking with 5% nonfat milk for 1 h at room temperature, the membranes were incubated overnight at 4°C with primary antibodies against HDAC2 (1:1000) and GAPDH (1:1000). Following washes, membranes were incubated with a horseradish peroxidase (HRP)‐conjugated secondary antibody (1:800) for 1 h at 37°C. Protein bands were visualized using an enhanced chemiluminescence (ECL) detection system and quantified by densitometry. GAPDH served as the loading control.

### 2.7. Cell Counting Kit‐8

The cytotoxicity of erythromycin and its optimal noncytotoxic concentration for subsequent experiments were determined using the Cell Counting Kit‐8 (CCK‐8; CK04, Dojindo Molecular Technologies, Kumamoto, Japan). RAW264.7 cells were seeded in 96‐well plates and treated with a range of erythromycin concentrations (0, 200, 400, 600, 800, and 1000 µg/mL) for 48 h. Following the treatment, 10 µL of CCK‐8 solution was added to each well, and the plates were incubated for an additional 2–4 h at 37°C. The absorbance at 450 nm was measured using a microplate reader. Cell viability was expressed as a percentage relative to the untreated control group (0 µg/mL), calculated according to the following formula:

Cell viability (%) = Atreatment−AblankAcontrol−Ablank × 100%,

Cell viability (%) = Acontrol−AblankAtreatment− Ablank × 100%, where Atreatment, Acontrol, and Ablank represent the absorbance values of the drug‐treated wells, untreated control wells, and culture medium‐only wells, respectively. The half‐maximal inhibitory concentration (IC50, µg/mL) was calculated from the dose–response curve.

### 2.8. Cellular Morphology Assessment by H&E Staining

To observe drug‐induced morphological changes in macrophages, RAW264.7 cells were seeded onto sterile glass coverslips placed in a 24‐well plate. After adherence, cells were treated with 10 µg/mL erythromycin (a concentration determined from the CCK‐8 assay to be noncytotoxic) or vehicle control for 48 h. Subsequently, the coverslips were carefully removed, and cells were fixed with 4% paraformaldehyde for 15 min at room temperature. Following fixation and washes, standard hematoxylin and eosin (H&E) staining was performed: nuclei were stained with hematoxylin, and cytoplasmic components were counterstained with eosin. The stained coverslips were then mounted on glass slides and observed under a light microscope for morphological evaluation.

### 2.9. Flow Cytometric Analysis of Macrophage Phenotypes

Following the designated treatments, cells were harvested and dissociated into single‐cell suspensions using trypsin (T1300; Solarbio, Beijing, China). After washing three times with cold phosphate‐buffered saline (PBS), approximately 1 × 10^6^ cells per sample were resuspended in staining buffer (PBS containing 2% FBS). Cells were then incubated for 30 min at 4°C in the dark with fluorescently conjugated antibodies against F4/80, CD206, and iNOS, according to the manufacturers′ recommendations. Appropriate unstained cells and isotype‐matched control antibodies were used to set gates and determine nonspecific background staining, respectively. After incubation, cells were washed twice with staining buffer to remove unbound antibodies, resuspended in 200 µL PBS, and analyzed immediately. Data acquisition was performed on a CytoFLEX flow cytometer (Beckman Coulter, Brea, CA, USA), and the percentages of M1 (F4/80^+^ iNOS^+^) and M2 (F4/80^+^ CD206^+^) macrophage subsets were determined using FlowJo software (Version 10.8.1, Tree Star).

### 2.10. Immunofluorescence Staining for *α*‐SMA

To assess the influence of macrophage phenotypes on fibroblast activation, a co‐culture system was established. Fibroblasts were co‐cultured with differentially polarized macrophages in transwell plates using DMEM complete medium supplemented with 10% fetal bovine serum. The fibroblast‐to‐macrophage ratio was 1:1. Macrophages were pre‐polarized as follows: M0 (naïve), M1 (stimulated with 100 ng/mL LPS for 24 h), and M2 (stimulated with 20 ng/mL interleukin‐4 for 24 h). The co‐culture groups were designated as: fibroblasts + M0, fibroblasts + M1, fibroblasts + M2, and fibroblasts + *a* 1:1 mixture of M1 and M2 macrophages. After 48 h of co‐culture, fibroblasts were fixed and processed for immunofluorescence staining. Briefly, cells were fixed with 4% paraformaldehyde, permeabilized with 0.1% Triton X‐100, blocked with 5% bovine serum albumin, and incubated overnight at 4°C with a primary antibody against *α*‐smooth muscle actin (α‐SMA). Following washes, cells were incubated with an appropriate fluorescent dye‐conjugated secondary antibody for 1 h at room temperature in the dark. Nuclei were counterstained with DAPI. Fluorescence images were captured using a fluorescence microscope, and *α*‐SMA expression levels were quantified based on integrated fluorescence intensity using ImageJ software (National Institutes of Health, Bethesda, MD, USA).

### 2.11. Statistical Analysis

Data are expressed as mean ± standard deviation (SD). Statistical analyses were conducted using SPSS software (version 13.0; IBM, Armonk, NY, USA). For comparisons among multiple groups, one‐way analysis of variance (ANOVA) was performed, followed by the Newman–Keuls post hoc test for specific pairwise comparisons. A two‐tailed *p*‐value of less than 0.05 was considered statistically significant. Graphs were generated using GraphPad Prism software (version 5.0; GraphPad Software, San Diego, CA, USA). Flow cytometry data were analyzed using FlowJo software (version 10.8.1; Tree Star, Ashland, OR, USA). Quantitative analysis of histopathological and immunofluorescence images was performed using ImageJ software (version 1.48; National Institutes of Health, Bethesda, MD, USA).

## 3. Results

### 3.1. Assessment of TS in Experimental Groups

**FIGURE 1:**
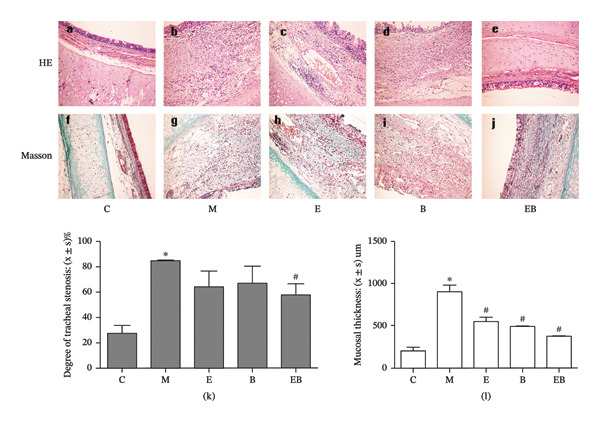
Histopathological evaluation of tracheal tissues. (a–e) Representative hematoxylin and eosin (H&E)‐stained sections of tracheal cross sections from the control group (C, a), model group (M, b), erythromycin‐treated group (E, c), budesonide‐treated group (B, d), and combination therapy group (EB, e). The control group shows a patent lumen and intact epithelium. The model group exhibits marked luminal narrowing and disrupted mucosal architecture. Treatment groups (E, B, EB) display better preservation of epithelial integrity and reduced luminal narrowing compared to group M. (f–j) Corresponding Masson’s trichrome‐stained sections from the same groups (C, f; M, g; E, h; B, i; EB, j). Collagen fibers are stained blue‐green. The control group shows staining primarily restricted to the cartilage. The model group reveals extensive collagen deposition (fibrosis) within the mucosa and submucosa. This fibrotic response is attenuated in all treatment groups, most profoundly in the EB group. (k) Quantitative analysis of tracheal stenosis severity (S). Data are mean ± SD; ^∗^ *p* < 0.05 vs. group C; ^#^ *p* < 0.05 vs. group M. (l) Quantitative analysis of the combined mucosa–submucosa thickness. Data are mean ± SD; ^∗^ *p* < 0.05 vs. group C; ^#^ *p* < 0.05 vs. group M.

A rabbit model of TS was successfully established, as evaluated on the 10th day post‐injury. Visual and histological inspection revealed a marked increase in the degree of tracheal narrowing in the model group (Group M) compared to the normal control group (Group C). Quantitative analysis of stenosis severity (S) confirmed this observation. Compared to Group M, the severity of TS was significantly reduced in all treatment groups: the budesonide group (Group B), the erythromycin group (Group E), and the combination therapy group (Group EB). The most pronounced amelioration was observed in Group EB (Figure 1k), where the reduction in stenosis severity reached statistical significance (*p* < 0.05).

### 3.2. Morphometric Analysis of Tracheal Wall Thickness

The combined thickness of the tracheal mucosa and submucosa was measured and compared across groups. A significant increase in thickness was observed in the model group (Group M) relative to the control group (Group C). In contrast, all treatment groups (B, *E*, and EB) exhibited a reduction in this parameter compared to Group M. Notably, the combination therapy group (EB) demonstrated the most pronounced reduction in wall thickness, which was significantly greater than the reductions observed in the single‐agent treatment groups (*p* < 0.05; Figure 1l).

### 3.3. Collagen Deposition Assessed by Masson’s Trichrome Staining

Masson’s trichrome staining was employed to evaluate collagen deposition within the tracheal wall (Figure 1f–j). In the control group (C), collagen staining (blue‐green) was primarily confined to the cartilage layer, with minimal staining in the mucosa and submucosa. In contrast, the model group (*M*) exhibited extensive and intense collagen deposition (blue‐green staining) throughout the mucosa and submucosa, indicative of substantial fibrosis. Treatment with either erythromycin (Group E) or budesonide (Group B) attenuated this fibrotic response, as evidenced by a visible reduction in collagen staining intensity and area. The most marked attenuation of collagen deposition was observed in the combination therapy group (EB).

### 3.4. mRNA Expression of Macrophage Phenotypic Markers

**FIGURE 2:**
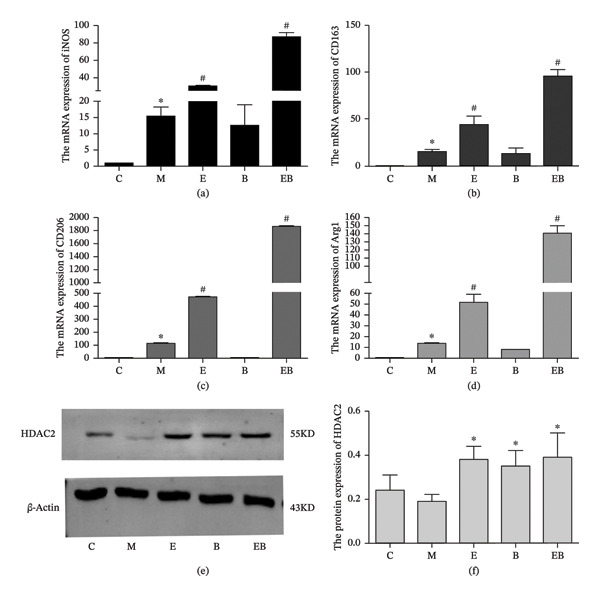
Real‐time PCR and Western blotting, a–d: The mRNA expression levels of iNOS, CD206, CD163, and Arg1 were increased in Group M (^∗^ *p* < 0.05 versus Group C), with the most pronounced increase in macrophage‐related gene expression observed in Group EB (^#^ *p* < 0.05 versus Group M). e–f: Compared to Group C, HDAC2 protein expression was decreased in Group M, whereas it was increased in Groups B, E, and EB, with the highest expression observed in Group EB (^#^ *p* < 0.05 vs. Group M).

The mRNA expression levels of macrophage phenotypic markers were quantified by qRT‐PCR (Figure 2). Compared to the control group (C), injury induction in the model group (*M*) led to a significant upregulation in the expression of both M1 (iNOS) and M2 (CD206, CD163, Arg1) macrophage‐associated genes. This confirmed the activation of a substantial immune response following tracheal scraping. In treatment groups, distinct patterns of gene expression modulation were observed. Notably, the mRNA levels of the M1 marker iNOS were most markedly elevated in the erythromycin (*E*) and combination therapy (EB) groups.

### 3.5. HDAC2 Protein Expression by Western Blot

HDAC2 protein expression was analyzed by western blot (Figure 2e–f). Compared to the control group (C), HDAC2 expression was significantly downregulated in the model group (*M*). All treatment interventions reversed this downregulation, with significant upregulation of HDAC2 observed in the budesonide (B), erythromycin (E), and combination therapy (EB) groups relative to group M (*p* < 0.05). Notably, the combination therapy group (EB) exhibited the highest level of HDAC2 protein expression among all groups.

### 3.6. Morphological Characterization of Macrophages Following Drug Treatment

**FIGURE 3:**
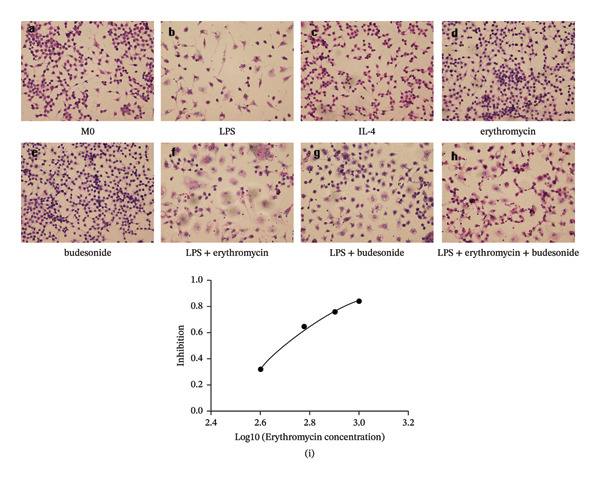
Morphological changes in macrophage cells, a–h: H&E staining showed that naïve macrophages (M0) were small and rounded. After stimulation with LPS, M0 macrophages differentiated into the M1 phenotype, becoming larger with extended pseudopodia and cytoplasmic vacuoles. Incubation with IL‐4 induced only minor morphological changes. Treatment with erythromycin (10 µg/mL) and budesonide resulted in a cell morphology similar to that observed in IL‐4‐treated cells. i: The cytotoxicity of erythromycin and its optimal noncytotoxic concentration for subsequent experiments were determined using the Cell Counting Kit‐8.

The morphological changes in RAW264.7 macrophages under various polarization conditions and drug treatments were assessed (Figure 3). Untreated, naïve macrophages (M0) displayed a small, rounded morphology. Stimulation with LPS induced a classical M1 phenotype, characterized by enlarged cell bodies, elongated pseudopodia, and increased cytoplasmic vacuolation. In contrast, stimulation with IL‐4 induced an alternative M2 phenotype, with cells remaining relatively small and rounded. Treatment with either erythromycin (10 µg/mL, a concentration based on reference) or budesonide alone induced morphological alterations resembling those of IL‐4‐induced M2 macrophages. Remarkably, even in the presence of the M1‐inducer LPS, co‐incubation with erythromycin or budesonide shifted macrophage morphology towards an M2‐like appearance.

### 3.7. Flow Cytometric Quantification of Macrophage Polarization

The polarization state of RAW264.7 macrophages under different conditions was assessed by flow cytometry using specific surface markers (Figure 4). In addition to the M2/M1 ratio, the absolute proportions of M1 and M2 macrophages were also analyzed. In the control group, the proportions of M1 and M2 macrophages were 37.36 ± 14.69 and 41.52 ± 6.70, respectively, with an M2/M1 ratio of 1.21 ± 0.36. In the LPS group, the corresponding values were 22.06 ± 0.24 and 21.17 ± 0.34, with an M2/M1 ratio of 0.96 ± 0.01. In the erythromycin group, the proportions of M1 and M2 macrophages were 27.19 ± 1.80 and 25.27 ± 1.75, respectively, with an M2/M1 ratio of 0.93 ± 0.00. In the budesonide group, the corresponding values were 35.56 ± 1.15 and 31.51 ± 1.16, with an M2/M1 ratio of 0.89 ± 0.00. In the combination treatment group, the proportions of M1 and M2 macrophages were 18.12 ± 1.02 and 16.73 ± 0.94, respectively, with an M2/M1 ratio of 0.92 ± 0.01. These findings indicate that erythromycin, budesonide, and their combination altered the distribution of macrophage subsets under the experimental conditions, supporting modulation of macrophage phenotypic balance rather than a uniform shift toward a canonical M2 phenotype.

FIGURE 4Proportion of M1 and M2 macrophages by flow cytometry.

**Figure:**
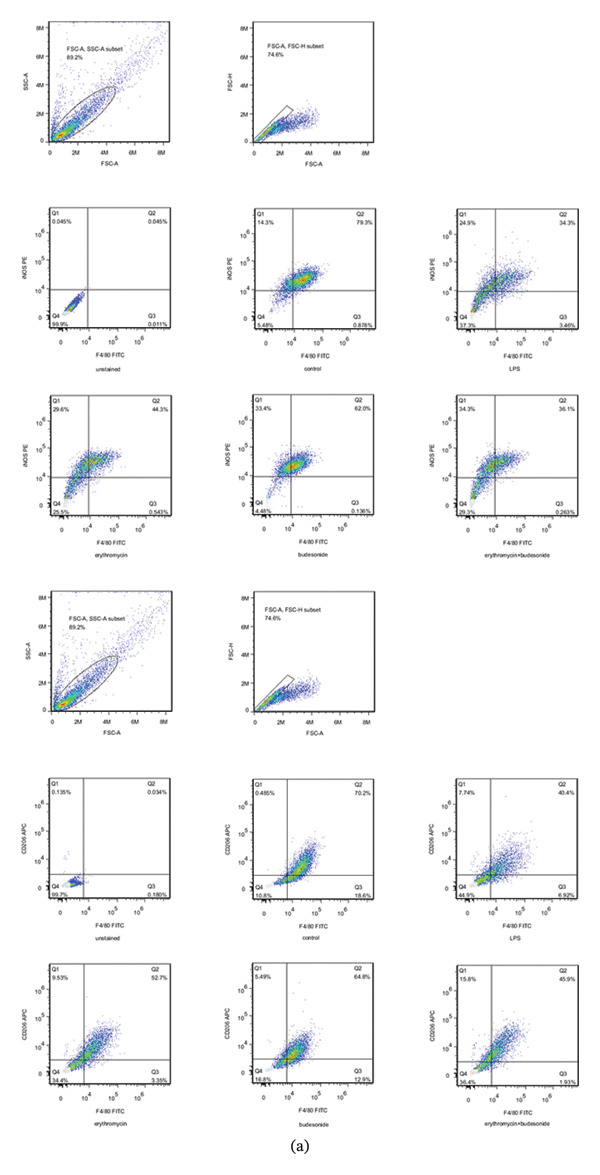

**Figure:**
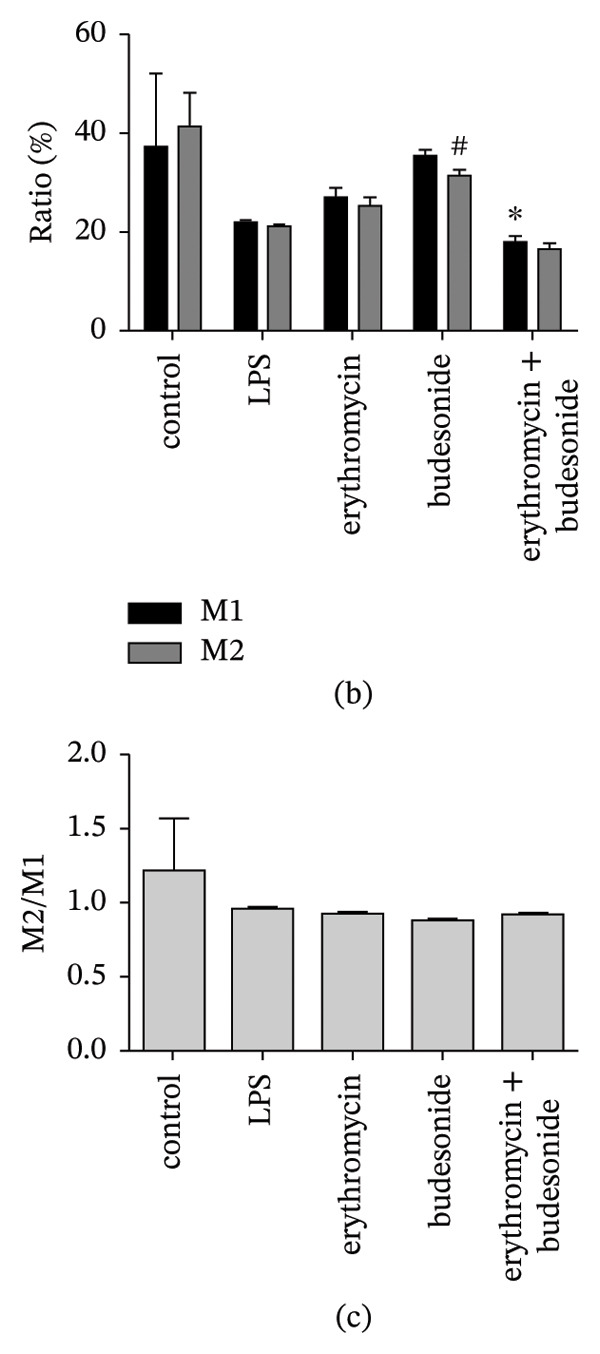

Absolute proportions of M1 and M2 macrophages, together with the corresponding M2/M1 ratios, were analyzed under different treatment conditions, showing altered macrophage subset distribution after treatment.

### 3.8. Macrophage Phenotype Modulates Fibroblast Activation in Co‐Culture

**FIGURE 5:**
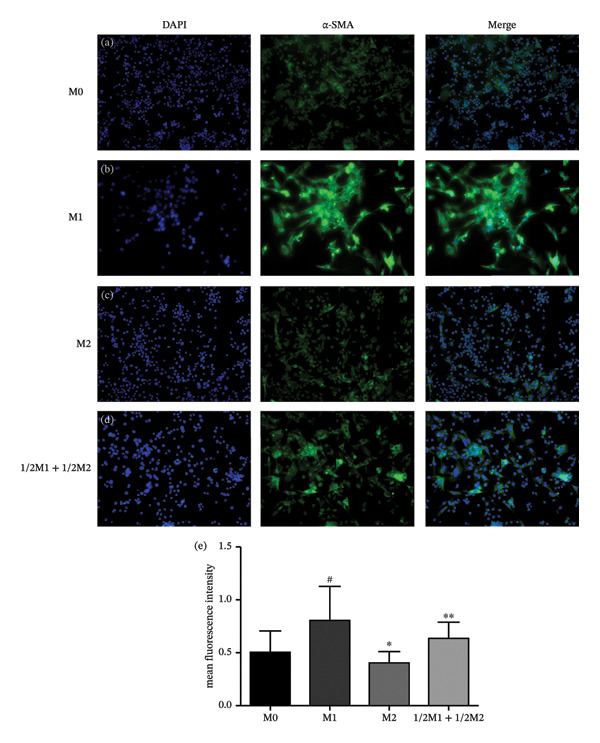
Effect of macrophage phenotype on fibroblast activation in co‐culture. Representative immunofluorescence images (a–d) and quantitative analysis (e) of *α*‐smooth muscle actin (α‐SMA) expression in fibroblasts following 48‐h co‐culture with differentially polarized macrophages. Fibroblasts were co‐cultured with: naïve macrophages (M0, a), M1‐polarized macrophages (M1, b), M2‐polarized macrophages (M2, c), or a 1:1 mixture of M1 and M2 macrophages (1/2M1+1/2M2, d). Nuclei are stained with DAPI (blue); *α*‐SMA is shown in red. Scale bar: 50 µm. (e) Quantitative analysis of *α*‐SMA fluorescence intensity. Data are presented as mean ± SD (*n* = 6). ^∗^ *p* < 0.05 vs. M0 group; ^#^ *p* < 0.05 vs. M1 group.

The functional impact of macrophage polarization on fibroblast activation was assessed by quantifying *α*‐SMA expression in a co‐culture system (Figure 5). Co‐culture with M1‐polarized macrophages significantly upregulated *α*‐SMA expression in fibroblasts compared to co‐culture with naïve (M0) macrophages (*p* < 0.05). In contrast, co‐culture with M2‐polarized macrophages resulted in a significant downregulation of *α*‐SMA compared to the M1 co‐culture condition (*p* < 0.05). Fibroblasts co‐cultured with a 1:1 mixture of M1 and M2 macrophages exhibited intermediate *α*‐SMA levels: significantly higher than those in the M0 or M2 co‐culture groups, yet significantly lower than in the M1 co‐culture group (*p* < 0.05).

## 4. Discussion

TS poses a significant clinical challenge, frequently emerging as a complication of endotracheal intubation or tracheotomy. Pathogenetically, TS is driven by aberrant wound healing, wherein an initial injury triggers a cascade of local and systemic inflammation. If dysregulated, this inflammatory response leads to excessive fibroblast proliferation and extracellular matrix deposition within the tracheal wall, culminating in fibrosis and luminal narrowing [3–5, 7–9]. A central event in this fibrotic process is the activation of tracheal mucosal fibroblasts by a milieu of inflammatory mediators, including transforming growth factor‐β1 (TGF‐β1) and vascular endothelial growth factor (VEGF), which collectively stimulate collagen synthesis and pathological tissue remodeling [6, 9]. In this study, we successfully recapitulated this pathophysiology using a rabbit model of TS. The model exhibited key histopathological features of human TS, including disruption of normal tracheal architecture, epithelial loss, and the formation of granulation tissue rich in proliferating fibroblasts and dense collagen fibers, as confirmed by Masson’s trichrome staining. This model mirrors the clinical etiology where mechanical trauma, ischemic pressure, and potential infectious stimuli converge to sustain a pro‐inflammatory state, characterized by elevated levels of cytokines such as IL‐1β, IL‐6, IL‐8, and TNF‐α [3–5, 7–9]. Such persistent inflammation compromises mucosal integrity, promotes apoptosis of ciliary cells, and ultimately drives the formation of the fibroproliferative tissue that constitutes the stenotic lesion [10]. Consistent with a central role for innate immunity, we found elevated expression of iNOS—a canonical marker of classically activated (M1) macrophages—in the injured tracheal tissue. This finding underscores the pivotal and sustained involvement of macrophage‐driven inflammation in the initiation and progression of TS.

Macrophages are master regulators of inflammation and repair, exhibiting remarkable plasticity that allows them to adopt diverse functional phenotypes in response to microenvironmental cues [11]. The classical M1 phenotype, induced by stimuli like interferon‐γ and lipopolysaccharide, is pro‐inflammatory and contributes to host defense and tissue destruction. In contrast, the alternative M2 phenotype, induced by cytokines such as IL‐4 and IL‐13, is associated with anti‐inflammatory responses, tissue repair, and fibrosis resolution [12]. The temporal transition from a predominant M1 response in the early injury phase to an M2‐dominated repair phase is critical for successful wound healing without excessive scarring. An imbalance, particularly a prolonged M1 or a dysregulated M2 response, can lead to chronic inflammation or pathological fibrosis, respectively [13, 14]. In the context of TS, previous work has identified an imbalance in macrophage polarization within stenotic tissues, with evidence of dysregulated and persistent macrophage presence [4]. Our data corroborates and extends this understanding. We observed a concurrent increase in mRNA expression of both M1 (iNOS) and M2 (CD163, CD206, Arg1) markers in the model group at day 10, suggesting a complex and potentially dysregulated immune landscape where both inflammatory and repair programs are activated but may be spatially or temporally misaligned, failing to effectively coordinate resolution.

The therapeutic modulation of macrophage polarization presents a promising strategy for intervening in fibrotic diseases. Macrolide antibiotics, notably erythromycin and azithromycin, have garnered attention for their potent immunomodulatory effects beyond their antimicrobial properties. These agents have been shown to favorably alter macrophage phenotype, promoting a shift toward the M2 state by enhancing the expression of anti‐inflammatory mediators while suppressing pro‐inflammatory markers [15–17]. Consistent with its reported clinical benefits in suppressing tracheal granulomas [3], our study found that erythromycin monotherapy improved tracheal histology, reduced stenosis severity, and was associated with changes in macrophage‐related marker expression. Interestingly, budesonide, a potent corticosteroid, appeared to broadly reduce macrophage marker expression, likely reflecting its generalized immunosuppressive effect on leukocyte recruitment and activation. While effective in reducing inflammation, this nonspecific suppression may not optimally orchestrate the repair process. The most striking finding emerged from the combination therapy: erythromycin and budesonide together produced synergistic therapeutic effects, achieving the greatest reduction in stenosis and fibrosis. This synergy suggests that the two drugs may act through partially complementary mechanisms, although the precise temporal and molecular interactions require further investigation.

An important finding of our study is that histone deacetylase 2 (HDAC2) expression was altered in parallel with the observed therapeutic and immunomodulatory effects. HDACs are key epigenetic enzymes that remove acetyl groups from lysine residues on histone tails and nonhistone proteins, thereby influencing chromatin compaction and gene transcription. The balance between histone acetyltransferase (HAT) and HDAC activity is a critical switch governing inflammatory gene expression [18, 19]. In respiratory diseases like COPD and asthma, reduced HDAC2 activity and expression are associated with amplified and steroid‐resistant inflammation [19, 20]. Our data revealed a significant downregulation of HDAC2 protein in the untreated TS model, mirroring a state of dysregulated epigenetic control that may permit sustained inflammatory gene expression. Crucially, treatment with erythromycin, budesonide, and most powerfully their combination, restored HDAC2 expression.

Previous studies have suggested that HDAC2 may participate in the regulation of inflammatory gene expression through epigenetic mechanisms, including modulation of transcription factor activity such as NF‐κB [18]. In the present study, HDAC2 expression was markedly reduced in the untreated TS model and was restored after treatment, particularly in the combination therapy group. This pattern, together with the observed changes in macrophage‐related markers, suggests that HDAC2 may be involved in the immunomodulatory effects associated with erythromycin and budesonide treatment. Notably, the concomitant alteration of both M1‐and M2‐related markers indicates that macrophage responses in TS may be more complex than a simple binary shift toward a canonical M2 phenotype. Therefore, our findings are better interpreted as reflecting modulation of macrophage phenotypic balance rather than a complete transition toward a single macrophage state.

Our in vitro experiments provide supportive evidence that erythromycin and budesonide can influence macrophage‐related phenotypic changes under experimental conditions. In RAW264.7 cells, combination treatment altered macrophage morphology and shifted the M2/M1 ratio toward 1.0, as assessed by flow cytometry. In the co‐culture system, fibroblast *α*‐smooth muscle actin (α‐SMA) expression varied according to macrophage phenotype, with lower expression observed in fibroblasts co‐cultured with M2‐polarized macrophages than in those co‐cultured with M1‐polarized macrophages. These findings suggest that macrophage phenotypic status may be associated with fibroblast activation and fibrotic remodeling in this model. However, the present data do not establish that HDAC2 directly mediates these effects in vivo. Because no HDAC2 inhibition, knockdown, knockout, or rescue experiments were performed, the mechanistic role of HDAC2 should be interpreted cautiously and requires further investigation.

Several limitations of this study should be acknowledged. First, the rabbit model used in this study was based on mechanical mucosal scraping and a 10‐day observation period. Therefore, it more closely reflects acute injury and early remodeling than fully established clinical fibrotic TS. Second, although changes in HDAC2 expression and macrophage‐related markers were observed in parallel with therapeutic improvement, the present study did not include HDAC2 inhibition, knockdown, knockout, or rescue experiments, and thus cannot establish a causal mechanistic role for HDAC2. Third, the sample size was relatively limited for mechanistic inference, and additional studies with more detailed temporal and functional analyses are warranted.

In conclusion, erythromycin combined with budesonide effectively attenuated injury‐induced TS and was associated with restoration of HDAC2 expression, changes in macrophage‐related markers, and reduced fibroblast activation in the experimental models used in this study. These findings support the potential therapeutic value of combination treatment in TS and suggest that HDAC2 may be involved in the underlying immunomodulatory process. Nevertheless, the current results support an association rather than a confirmed causal mechanistic pathway. Further studies incorporating direct functional manipulation of HDAC2 will be required to clarify whether HDAC2 directly regulates macrophage phenotypic changes and contributes to the anti‐fibrotic effects observed in vivo.

## Funding

This work was supported by China National Natural Science Foundation (grant numbers 81760001 and 82060003).

## Disclosure

A preprint has previously been published: Jinghua Gan, Guangnan Liu, 2023 [21].

## Ethics Statement

All animal experiments were conducted according to the Principles of Laboratory Animal Care (National Society for Medical Research, Permit Nos. KY‐0048 and KY 0049). The study protocol was approved by the Second Affiliated Hospital of Guangxi Medical University.

## Conflicts of Interest

The authors declare no conflicts of interest.

## Data Availability

The data that support the findings of this study are available from the corresponding author upon reasonable request.
